# Automatic heart volume measurement from CMR images using ant colony optimization with iterative salient isolated thresholding

**DOI:** 10.1186/1532-429X-14-S1-P286

**Published:** 2012-02-01

**Authors:** El-Sayed Ibrahim, Shannon Birchell, Sherif Elfayoumy

**Affiliations:** 1 Department of Radiology, University of Florida, Jacksonville, FL, USA; 2 School of Computing, University of North Florida, Jacksonville, FL, USA

## Summary

Manual segmentation of CMR images is inefficient and inconsistent method for measuring ventricular volumes. In this study, a new technique (ACOISIT) is developed and implemented for measuring ventricular volumes. The technique is based on automatic delineation of blood-myocardium border using ant colony optimization with salient isolated thresholding. The technique was implemented on datasets from eight volunteers and the results were compared to manual segmentation. ACOISIT showed good agreement with the gold standard and was faster and more consistent than manual segmentation.

## Background

Ventricular volumes are usually measured from cardiovascular magnetic resonance (CMR) images through manual delineation of the left and right ventricles (LV, RV) endocardial contours in parallel short-axis images. However, manual segmentation consumes long processing time and reduces measurement consistency. In this study, a new artificial intelligence technique (ant colony optimization with iterative salient isolated thresholding, ACOISIT) is developed and implemented for measuring ventricular volumes through automatic delineation of blood-myocardium border.

## Methods

Eight volunteers were scanned on a 3.0T-Siemens scanner to acquire stack of cine short-axis images covering both ventricles. The ACOISIT technique was implemented on all images to delineate LV and RV endocardial contours, from which ventricular volumes were measured using Simpson’s approximation, and compared to manual segmentation (gold standard). Fig.1 describes the ACOISIT algorithm, which is composed of iterative implementation of two modules: adaptive thresholding segmentation (minimum entropy) and edge detection. The ACOISIT technique is based on actual ant colonies by simulating the adaptive process by which ants find food and transfer it to their nest, which ultimately leads to the shortest path between food source and the nest. Path weights (pheromones) are probabilistically assigned, and ants move in different directions until a boundary pixel is found, which is then added to the solution set. The process iterates until the termination criterion is met. Statistical t-test and Bland-Altman analysis were conducted to test the ventricular volume differences between ACOISIT and manual segmentation.

## Results

Fig.2 illustrates the ACOISIT technique and shows segmentation results of different cases with various degrees of complexity. Processing time was 6 minutes per dataset for both LV and RV on Intel dual-core 1.73 MHz personal computer (about 30 minutes by manual segmentation). The t-test showed non-significant ventricular volume differences by ACOISIT and manual segmentation (p>0.1). Bland-Altman analysis showed no bias between volume measurements as all differences lied within the 2 standard-deviations (SD) agreement limit. Mean±SD for LV and RV volume differences were 3.8±10.1 ml and -2.1±14.2 ml. Corresponding correlation coefficients were 0.93 and 0.91.

## Conclusions

LV and RV volume measurements by ACOISIT are in good agreement with the gold standard. The integration of salient thresholding segmentation in ACOISIT resolved ambiguous regions and led to accurate boundary detection. The ACOISIT technique is significantly faster (parallel processing by different intelligent agents (ants)) and more consistent than manual segmentations. The technique provides both LV and RV segmentations in a single processing, and provides a stability measure to gauge the solution integrity. The technique detects endocardial borders in the case of severe trabeculations, which is a challenging task for automatic segmentation.

## Funding

N/A

